# Association between water, sanitation, and hygiene (WASH) practices and childhood stunting in low- and middle-income countries: a systematic review and meta-analysis

**DOI:** 10.1186/s12889-026-27639-z

**Published:** 2026-05-05

**Authors:** Frengki Prabowo Saputro Wijayanto, Artha Maressa Theodora Simanjuntak, Jesslyn Chang, Muhammad Miftahul Asrar, Annamaria Phoebe Swastidharmistha, Rais Amaral Haq, Made Aditya Krisnanta Gandhy, Mohammad Zaky Rabih, Muhammad Gilang Dwi Putra, Rodman Tarigan Girsang

**Affiliations:** 1 Child Health Department, Faculty of Medicine, Universitas Padjadjaran, Bandung, West Java 40161 Indonesia; 2 Medical Education Study Program, Faculty of Medicine, Public Health, and Nursing, Gadjah Mada University, Sleman, Yogyakarta, 55281 Indonesia; 3 Medical Education Study Program, Faculty of Medicine, Lampung University, Bandar Lampung, Lampung 35141 Indonesia

**Keywords:** Children, Low- and middle-income countries, Stunting, WASH, Meta-analysis

## Abstract

**Introduction:**

Childhood stunting remains a major public health challenge in low- and middle-income countries (LMICs). Inadequate water, sanitation, and hygiene (WASH) conditions contribute to recurrent infections and chronic undernutrition; however, evidence regarding which specific WASH components most strongly influence stunting remains inconsistent. This study aimed to systematically evaluate the association between WASH practices and childhood stunting in LMICs.

**Methods:**

We conducted a systematic review and meta-analysis following PRISMA 2020 guidelines. PubMed, Scopus, Web of Science, Cochrane Library, Epistemonikos, EBSCOhost, and Google Scholar were searched up to July 2025. Observational studies from LMICs assessing associations between WASH practices and childhood stunting were included. Study quality was evaluated using the Newcastle–Ottawa Scale. Random-effects meta-analyses were used to pool odds ratios (ORs) with 95% confidence intervals, with publication bias and sensitivity analyses performed.

**Results:**

Sixteen studies involving 36,574 children were included. Improved water quality (OR = 0.39; 95% CI: 0.18–0.87) and water accessibility (OR = 0.69; 95% CI: 0.49–0.98) were significantly associated with lower odds of stunting, whereas drinking water source and household water treatment were not. Among sanitation indicators, improved sanitation facility type (OR = 0.71; 95% CI: 0.53–0.94) and environmental sanitation (OR = 0.05; 95% CI: 0.00–0.64) were significantly associated with lower odds of stunting, while toilet access, safe disposal of child feces, and garbage disposal showed no consistent associations. Hygiene practices were also associated with lower odds of stunting, particularly hand hygiene (OR = 0.54; 95% CI: 0.30–0.98) and family hygiene (OR = 0.40; 95% CI: 0.21–0.74), whereas general hygiene was not significantly associated with stunting. Substantial heterogeneity was observed across several outcomes.

**Conclusions:**

Several WASH components were associated with lower odds of childhood stunting in LMICs, with more consistent evidence for drinking water quality, water accessibility, and sanitation facility type. Findings for other components were inconsistent. Given substantial heterogeneity and the observational nature of included studies, these results should be interpreted cautiously, and further research is needed to establish causal relationships.

**Trial registration:**

PROSPERO CRD420251145998.

**Supplementary Information:**

The online version contains supplementary material available at 10.1186/s12889-026-27639-z.

## Introduction

Stunting remains a major public health challenge globally. Low- and middle-income countries (LMICs) bear the overwhelming majority of the stunting burden, accounting for 98% of all stunted children globally [1]. United Nations of Children’s Fund (UNICEF) reported 23.2% of children under the age of five were stunted globally in 2024 [2]. A recent study emphasized 22% of children under five are affected by stunting, particularly in the South and Southeast Asia region, with low gross national income (GNI) rates [3, 4]. Stunting has long-term effects on individuals, including diminished cognitive and physical development, which was later associated with poor health and degenerative diseases [5]. Its causative-relationship on cognitive development and educational attainment which leads to lower adult income and productivity. The World Bank estimates stunting may reduce up to 7% of countries’ gross domestic product (GDP) per capita [6].

Childhood stunting arises from multiple interrelated factors, including insufficient nutrition, poor hygiene, and repeated infectious exposures. Poor hygiene and sanitary conditions in the households may result in chronic exposure to environmental pathogens, which may lead to alteration in gut microbiota [7, 8]. Malnutrition caused by changes in the gut microbiota morphology and function may provoke stunting among children [9]. Poor sanitations enable the transmission of faecal-oral pathogens and is a risk factor for numerous adverse health outcomes, including gastrointestinal diseases, undernutrition, and stunting [10]. Water access is a critical factor for determining stunting prevalence [11]. A recent study reported a 3.3 times higher risk of stunting in children with contaminated drinking water [12]. Additionally, inadequate water access and proper sanitation could contribute to children’s poor cognitive development in stunted children [13].

Water, sanitation, and hygiene (WASH) research have shown evidence that good practices are central to the realization of the global elimination of stunting in children [14, 15]. A multicenter, cross-sectional study consisting of 94 LMICs suggested focusing on younger children is key to optimizing the impact of programmes that aim to prevent undernutrition and infections, which thereby reduce stunting [16]. This issue is particularly important in vulnerable populations, as children in LMICs are often exposed to low levels of early stimulation, greater socioeconomic deprivation, and persistent health challenges [17].

Despite its importance, secondary research on WASH-related factors in children with stunting remains fragmented. While previous meta-analyses have primarily evaluated the impact of WASH interventions combined with nutrition on child growth outcomes, their findings have been inconsistent and have largely focused on intervention-based effects rather than specific WASH exposures, thereby limiting the ability to isolate the independent contribution of individual WASH components [18]. In contrast, the present study focuses exclusively on WASH-related factors without combining them with nutritional interventions, aiming to better understand the independent associations between specific WASH indicators and childhood stunting. In light of these global concerns, adding high-quality meta-analyses are urgently needed to address these problems in an appropriate manner. This study aims to determine the association between WASH practices on childhood stunting in LMICs.

## Method

### Study design

This study was designed as a systematic review and meta-analysis, conducted in accordance with the PRISMA guidelines 2020 [19]. The protocol was registered in PROSPERO (registration number: CRD420251145998).

### Study eligibility criteria

Studies were eligible for inclusion if they involved children residing in LMICs and evaluated the association between WASH-related factors and stunting, with sufficient data on the number of stunting events to allow comparison across different levels of WASH exposure. Both primary studies and analyses based on existing datasets, such as national surveys or previously collected data, including Demographic and Health Surveys (DHS) and Multiple Indicator Cluster Surveys (MICS) were included. Exclusion criteria comprised secondary research articles (e.g., systematic reviews, meta-analyses, editorials, and commentaries), case reports, and studies for which the full text could not be retrieved despite two follow-up attempts to the corresponding authors.

### Search strategy and selection process

A systematic literature search was conducted up to July 2025 in PubMed, Scopus, Web of Science, the Cochrane Library, Epistemonikos, and databases accessed via the EBSCOhost platform (including Academic Search Complete, MEDLINE with Full Text, and Business Source Complete). Additionally, we conducted a systematic search in Google Scholar and screened the first 300 results sorted by relevance. The search strategy combined keywords related to water, sanitation, hygiene, children, and stunting were aligned with Medical Subject Headings (MeSH), as detailed in Supplementary File 1. No restrictions on year of publication and language were applied. All identified records from 5 databases and EBSCOhost were imported into Rayyan.ai for screening and duplicate removal [19]. Two reviewers (AMTS and FPSW) screened titles and abstracts based on predefined eligibility criteria, followed by full-text assessment. Discrepancies were resolved through discussion with a third reviewer (RTS). For Google Scholar, two reviewers (JC and MMA) independently screened titles and abstracts. Potentially eligible records were exported to a spreadsheet, where duplicates were removed prior to full-text assessment. Discrepancies were resolved through discussion with a third reviewer (RTS).

### Data extraction and quality assessment

Data from each included study were systematically extracted and summarized in a standardized table, including the first author and year of publication, study design, country, sample size, sex distribution (male/female), and participants’ age. Data extraction was independently performed by two reviewers (FPSW and AMTS), with accuracy verified by a third reviewer (MMA). Statistical analyses were conducted by FPSW. Additionally, the number of stunting events was extracted from each study.

The methodological quality of the included studies was assessed using the Newcastle–Ottawa Scale (NOS). For case-control studies, the original NOS was applied as intended. Because many included studies were cross-sectional, an adapted version of the NOS was applied for these studies, consistent with previous systematic reviews [20]. The adapted NOS evaluated three domains: (1) selection (representativeness of the sample and ascertainment of exposure), (2) comparability (control for potential confounders), and (3) outcome (assessment of outcome and appropriateness of statistical analysis). Each study was assigned a score based on these domains, and overall study quality was categorized as good, fair, or poor. Two reviewers (MAKG and JC) independently performed the quality assessments, and any discrepancies were resolved through discussion with a third reviewer (APS). The final NOS ratings were summarized in tabular form to facilitate comparison and enhance interpretability across studies.

### Outcome endpoints

In the water domain, water quality referred to the safety of drinking water, including microbiological and physical characteristics, and was categorized as “safe” or “unsafe”. Water accessibility reflected the availability and physical access to water, based on distance to the source, time required for collection, or reported availability, and was categorized as access or no access. Water source was defined as the type and origin of water used for drinking and was categorized as “improved” or “unimproved” according to study-specific classifications. Water treatment described any household-level method used to improve water safety prior to consumption and was categorized as “treated” or “untreated”.

In the sanitation domain, sanitation facility type was defined as the type of toilet or latrine used by households and was categorized as “improved” or “unimproved” according to study-specific classifications. Environmental sanitation represented overall household sanitation conditions, typically assessed using composite indicators capturing multiple aspects of the domestic environment (e.g., waste, drainage, and cleanliness), and was categorized as “good” or “poor”. Toilet access described the availability or use of sanitation facilities within the household and was categorized as “access” or “no access”, including distinctions such as private, shared, or absent facilities when reported. Safe disposal of feces was defined as the method used for the disposal of human feces, particularly those of children, and was categorized as “safe” (e.g., disposal into a toilet or latrine) or “unsafe” (e.g., disposal into open environments, including fields, bushes, open water bodies, disposal with household waste, or leaving feces on the ground). Garbage disposal referred to the management of solid household waste and was categorized as “safe” or “unsafe” based on whether waste was disposed of through safe and controlled methods or left in open or unmanaged environments.

In the hygiene domain, hand hygiene referred to handwashing behavior and practices, particularly the use of soap at critical times such as before feeding and after defecation, and was categorized as “yes” or “no”. Family hygiene referred to hygiene behaviors practiced within the household, including caregiver and child hygiene practices, and was categorized as “good” or “poor”. General hygiene practices referred to overall household hygiene conditions, often measured using composite indicators that capture multiple hygiene-related behaviors and environmental cleanliness, and was categorized as “good” or “poor”.

### Statistical analysis

Statistical analyses were conducted using RStudio (version 4.4.1) with the “meta” package. Pooled odds ratios (ORs) with 95% confidence intervals (CIs) were calculated using the Mantel-Haenszel method with a random-effects model. Effect estimates were derived from the number of events and total participants in the exposed and comparison groups reported in each study. Therefore, the pooled estimates represent crude odds ratios based on event-count data rather than adjusted effect estimates from multivariable models. In the presence of zero events in one study arm, a continuity correction was applied to enable effect estimation. Studies with zero events in both arms would be excluded from odds ratio calculations, as they do not contribute information on relative effects [21]. When sparse data were substantial, alternative methods such as the Peto odds ratio or generalized linear mixed models may be considered [22]. Statistical significance was defined as a p-value < 0.05. Between-study heterogeneity was assessed using the *I*^*2*^ statistic, with *τ*^*2*^ estimated using the restricted maximum likelihood (REML) method. Values ≤ 50% indicated low heterogeneity and > 50% indicated substantial heterogeneity [23]. Publication bias was evaluated by funnel plots when ≥ 10 studies were included, complemented by Egger’s regression test. For meta-analyses including fewer than 10 studies, publication bias was assessed using the DOI plot and Luis Furuya-Kanamori (LFK) index [24]. Sensitivity analyses were performed to assess the robustness of the pooled estimates. Subgroup analyses were performed for outcomes with more than three studies, while meta-regression analyses were conducted when more than ten studies were available.

## Result

### Study selection

**Fig. 1:**
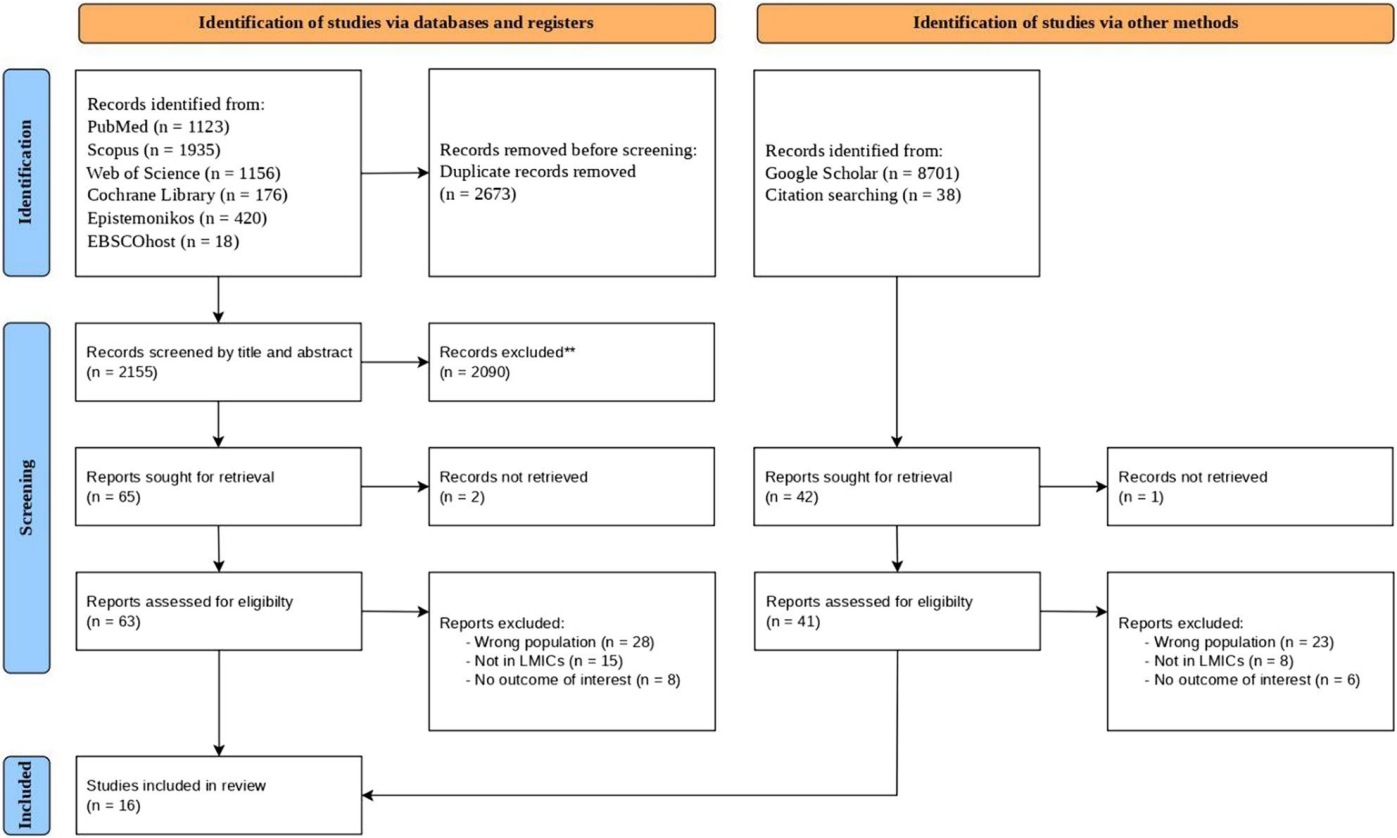
Diagram flowchart of selection process. **did not meet inclusion criteria

A total of 4,828 records were identified from PubMed, Scopus, Web of Science, the Cochrane Library, Epistemonikos, and databases accessed via the EBSCOhost platform (including Academic Search Complete, MEDLINE with Full Text, and Business Source Complete). After removing 2,673 duplicates, 2,155 records were screened by title and abstract, of which 2,090 were excluded for not meeting the inclusion criteria. Two additional studies could not be retrieved. Sixty-three full texts were assessed for eligibility, and 51 were excluded due to wrong population, non-LMIC setting, and no outcome of interest. In addition, 870 records were identified through Google Scholar and 38 through citation searching. Of these, one could not be retrieved, and 41 full texts were assessed, with 37 excluded for the same reasons. Ultimately, 16 studies met the eligibility criteria and were included in the meta-analysis. The study selection process is summarized in the PRISMA flowchart Fig. 1.

### Characteristic of included studies

A total of 36,574 children were evaluated for WASH practices and their association with stunting. The included studies employed cross-sectional and case-control designs and were published between 2016 and 2025 across several countries, including Ethiopia, Indonesia, Mozambique, and Bangladesh. Study populations were drawn from both urban and rural areas, with settings including community-based and health center-based environments. The included studies involved children aged 0–59 months. Detailed characteristics of the study populations are presented in Table 1.

**Table 1:** Characteristics of included studies

Study	Study design	Country	Residence	Setting	Population	M/F	Age (month)
Ademas et al., 2021 [25]	Cross-sectional	Ethiopia	Urban	Community	630	302/328	30.94 ± 14.6
Ahmadi et al., 2020 [26]	Cross-sectional	Indonesia	Rural	Community	82	NR	NR
Ainy et al., 2021 [27]	Cross-sectional	Indonesia	Urban	Community	393	202/191	33.8 ± 16.3
Braun et al., 2024 [10]	Cross-sectional	Mozambique	Urban	Community	789	389/400	NR
Ghosh et al., 2021 [28]	Cross-sectional	Bangladesh	Rural	Community	108	55/53	NR
Hasan et al., 2023 (MICS) [29]	Cross-sectional	Bangladesh	Urban: 4160 (21%) Rural: 18,361 (79%)	Community	22,521	11,654/10,867	29.6 ± 17
Hasan et al., 2023 (BDHS) [29]			Urban: 2758 (26.6%) Rural: 5299 (73.4%)	Community	7993	4202/3855	29 ± 17.1
Hasanah et al., 2020 [30]	Case-control	Indonesia	Urban	Community	150	NR	NR
Ilahi et al., 2022 [31]	Case-control	Indonesia	Urban	Community	106	NR	NR
Khaerani et al., 2025 [32]	Case-control	Indonesia	Rural	Health Center	56	23/33	25.7 ± 13.9
Novianti et al., 2023 [11]	Case-control	Indonesia	Rural	Health Center	212	NR	NR
Pradana et al., 2023 [33]	Case-control	Indonesia	Rural	Health Center	50	26/24	32 ± 14.3
Rah et al., 2018 [34]	Cross-sectional	Indonesia	Rural	Community	1450	739/711	19.5 ± 8.6
Sapriansyah et al., 2024 [35]	Case-control	Indonesia	Rural	Health Center	120	50/70	32.2 ± 13.1
Sari et al., 2024 [36]	Cross-sectional	Indonesia	Urban	Health Center	133	66/67	23.4 ± 16.2
Torlesse et al., 2016 [37]	Cross-sectional	Indonesia	Rural	Community	1366	681/685	11.9 ± 6.3
Woldesenbet et al., 2024 [38]	Cross-sectional	Ethiopia	Rural	Community	415	204/211	43 ± 9.5

The data were presented using mean ± SD

*MICS*  Multiple Indicator Cluster Survey, *BDHS*  Bangladesh Demographic and Health Survey, *M*  Male, *F*  Female, *NR*  Not Reported

### Quality assessment

The methodological quality of the included studies was assessed using the NOS, adapted for cross-sectional and case–control designs. Overall, the quality of the included studies was generally high. Among the 16 included studies, the majority were rated as good quality (*n* = 14), with total scores ranging from 7 to 9. Specifically, all case–control studies achieved good quality ratings, with scores between 7 and 9, reflecting strong performance in the selection and outcome domains, although some variability was observed in comparability due to limited adjustment for confounding factors. For cross-sectional studies, most were also classified as good quality (scores = 7), indicating adequate methodological rigor in selection and outcome assessment. However, two studies (Ahmadi et al., 2020; Sapriansyah et al., 2024) were rated as fair quality, primarily due to weaker comparability domains and limited control for potential confounders [26, 35].(Table 2) Across studies, the selection domain consistently received high scores, suggesting appropriate sampling strategies and representativeness of study populations. The outcome/exposure assessment domain was also generally robust. In contrast, the comparability domain showed the greatest variability, indicating that adjustment for confounding factors was not uniformly applied across studies.

**Table 2:** Quality assessment of included studies using NOS

Author, year	Study design	Selection	Comparability	Outcome	Total Score	Category
Ademas et al., 2021 [25]	Cross-sectional	★★★	★★	★★	7	Good
Ahmadi et al., 2020 [26]	Cross-sectional	★★★		★★	5	Fair
Ainy et al., 2021 [27]	Cross-sectional	★★★	★★	★★	7	Good
Braun et al., 2024 [10]	Cross-sectional	★★★	★★	★★	7	Good
Ghosh et al., 2021 [28]	Cross-sectional	★★★	★★	★★	7	Good
Hasan et al., 2023 (MICS) [29]	Cross-sectional	★★★	★★	★★	7	Good
Hasanah et al., 2020 [30]	Case-control	★★★★	★★	★★★	9	Good
Ilahi et al., 2022 [31]	Case-control	★★★★	★	★★★	8	Good
Khaerani et al., 2025 [32]	Case-control	★★★★		★★★	7	Good
Novianti et al., 2023 [11]	Case-control	★★★★	★★	★★★	9	Good
Pradana et al., 2023 [33]	Case-control	★★★★	★	★★★	8	Good
Rah et al., 2018 [34]	Cross-sectional	★★★	★★	★★	7	Good
Sapriansyah et al., 2024 [35]	Case-control	★★★★		★★	6	Fair
Sari et al., 2024 [36]	Cross-sectional	★★★	★★	★★	7	Good
Torlesse et al., 2016 [37]	Cross-sectional	★★★	★★	★★	7	Good
Woldesenbet et al., 2024 [38]	Cross-sectional	★★★	★★	★★	7	Good

### Forest plot of the association between WASH practices and childhood stunting

#### Association between water practices and childhood stunting

**Fig. 2:**
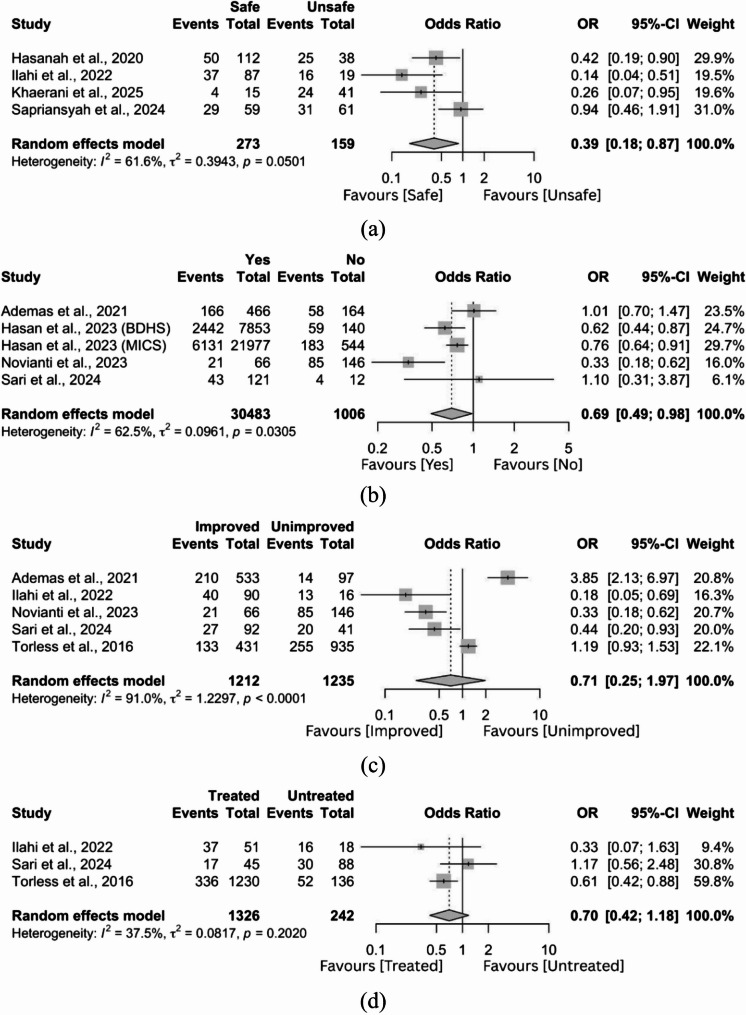
Association between drinking water quality (**a**), water accessibility (**b**), drinking water source (**c**), and water treatment (**d**) and childhood stunting

The association between water practices and childhood stunting is presented in the forest plot (Fig. 2). The pooled analysis showed that improved water quality was significantly associated with lower odds of childhood stunting (OR = 0.39, 95%CI: 0.18–0.87; *p* = 0.0212), with substantial heterogeneity (*I*^*2*^ = 61.6%). Similarly, water accessibility was significantly associated with lower odds of stunting (OR = 0.69, 95%CI: 0.49–0.98; *p* = 0.0385), with substantial heterogeneity (*I*^*2*^ = 62.5%). In contrast, no significant associations were observed for drinking water source and water treatment with childhood stunting (OR = 0.71; 95%CI: 0.25–1.97; *p =* 0.5061; *I*^*2*^ = 91.0%) and (OR = 0.70; 95%CI: 0.42–1.18; *p =* 0.1817; *I*^*2*^ = 37.5%), respectively.

#### Association between sanitation practices and childhood stunting

**Fig. 3:**
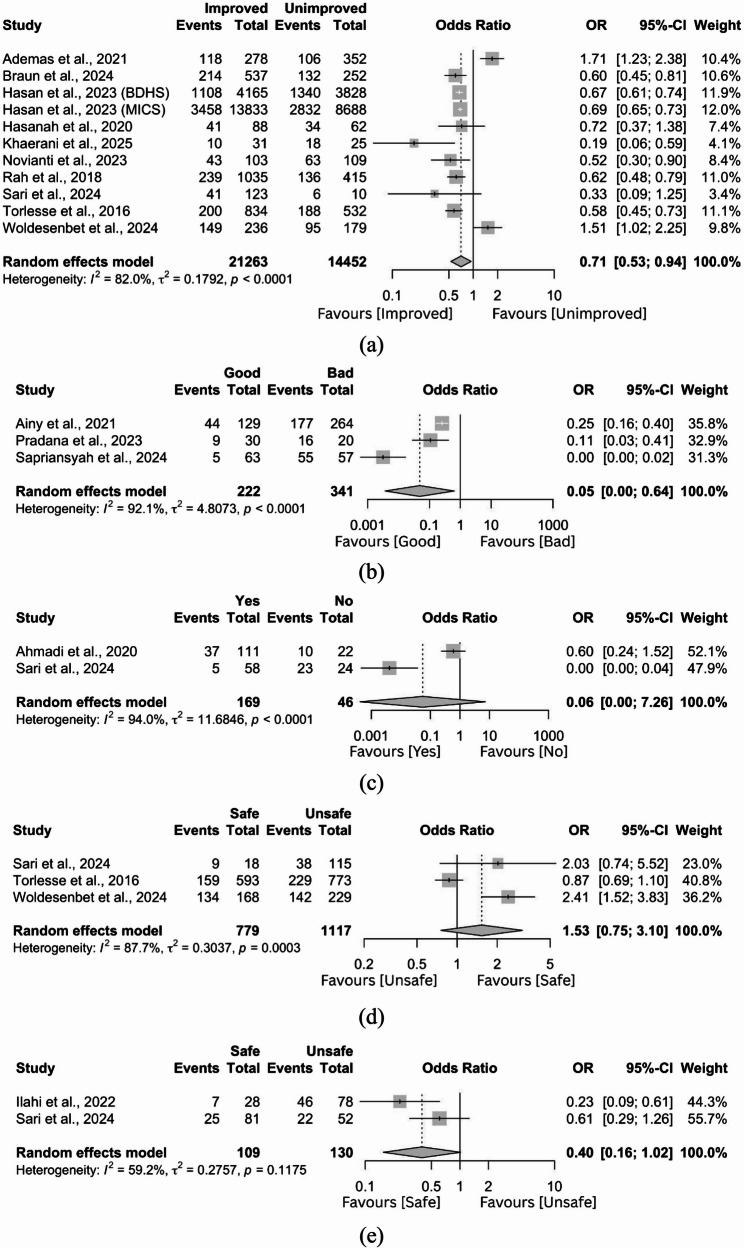
Association between sanitation facility type (**a**), environmental sanitation (**b**), toilet access (**c**), safe disposal of feces (**d**), and garbage disposal (**e**) with childhood stunting

The association between sanitation practices and childhood stunting is presented in the forest plot (Fig. 3). The analysis showed that improved sanitation facility type was significantly associated with lower odds of childhood stunting (OR = 0.71, 95%CI: 0.53–0.94; *p =* 0.0175), with substantial heterogeneity (*I*^*2*^ = 82.0%). Similarly, improved environmental sanitation was significantly associated with lower odds of childhood stunting (OR = 0.05, 95%CI: 0.00–0.64; *p =* 0.0215), with substantial heterogeneity (*I*^*2*^ = 92.1%). In contrast, toilet access, safe disposal of feces, and garbage disposal were not significantly associated with lower odds of childhood stunting (OR: 0.06; 95%CI: 0.00–7.26; *p =* 0.2444; *I*^*2*^ = 94%), (OR: 1.53; 95%CI: 0.75–3.10; *p =* 0.2387; *I*^*2*^ = 87.7%), and (OR: 0.40; 95%CI: 0.16–1.02; *p =* 0.0540; *I*^*2*^ = 59.2%), respectively.

#### Association between hygiene practices and childhood stunting

**Fig. 4:**
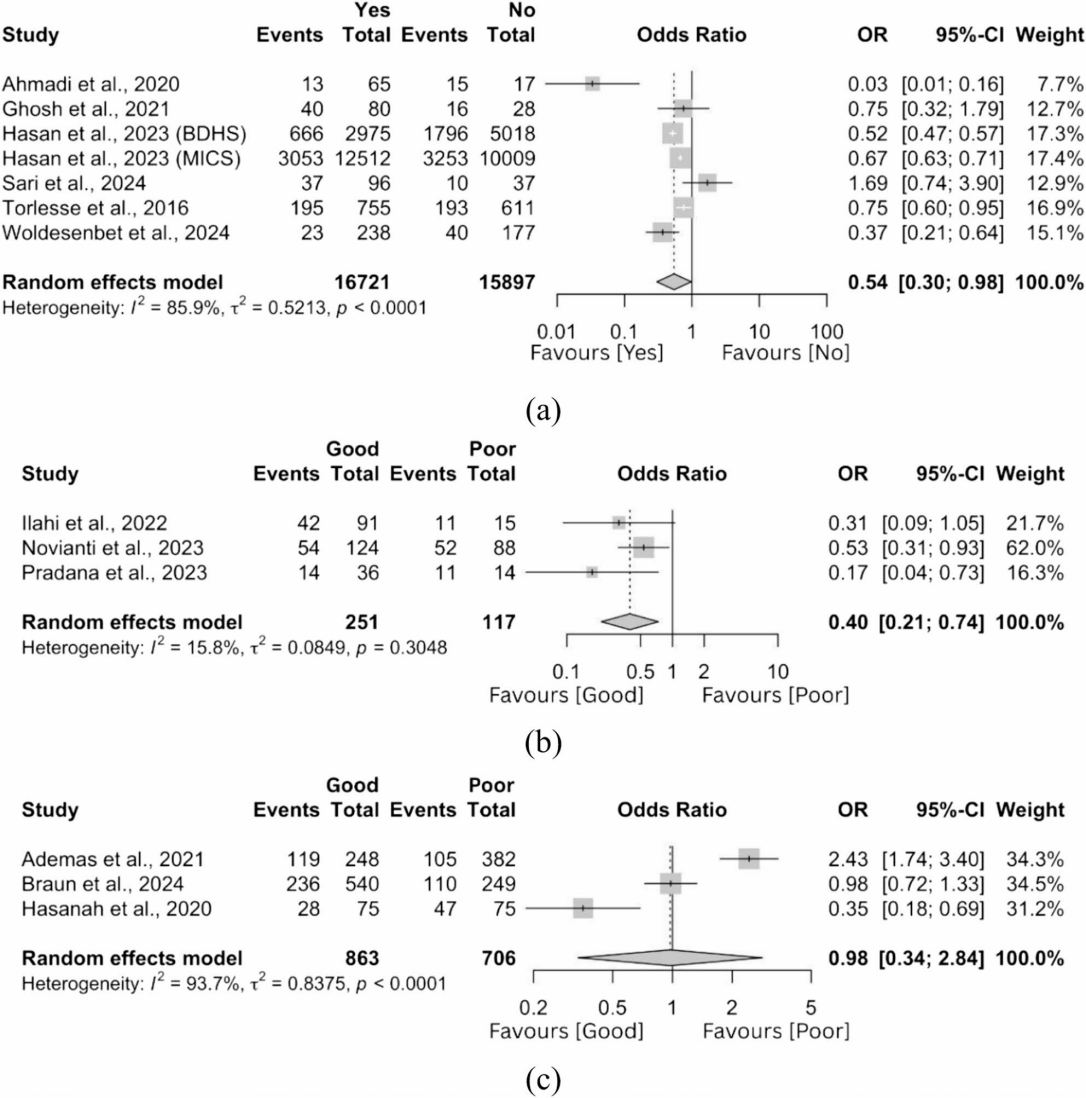
Association between hand hygiene (**a**), family hygiene (**b**), and general hygiene practices (**c**) and childhood stunting

The association between hygiene practices and childhood stunting is presented in the forest plot (Fig. 4). The analysis showed that hand hygiene was significantly associated with lower odds of childhood stunting (OR = 0.54; 95%CI: 0.30–0.98; *p* = 0.0422), with substantial heterogeneity (*I*^*2*^ = 85.9%). Similarly, family hygiene was associated with lower odds of childhood stunting (OR = 0.40; 95%CI: 0.21–0.74; *p =* 0.0037), with no heterogeneity (*I*^*2*^ = 15.8%). In contrast, general hygiene practices were not significantly associated with lower odds of childhood stunting (OR = 0.98; 95%CI: 0.34–2.84; *p =* 0.9631), with substantial heterogeneity (*I*^*2*^ = 93.7%).

### Subgroup and meta-regression analysis

Subgroup analysis is presented in Supplementary Table 2. For water-related outcomes, drinking water quality was significantly associated with lower odds of stunting in urban settings, community-based studies, and studies of good methodological quality, whereas no significant associations were observed in rural settings, health center–based studies, and studies of fair quality. Water accessibility was also significantly associated with lower odds of stunting across several subgroup categories, including case-control and cross-sectional study designs, studies conducted in Bangladesh, and in rural, mixed (urban and rural), and community-based settings. In contrast, no significant associations were observed in studies conducted in Ethiopia and Indonesia, as well as in urban populations and health center-based settings. Drinking water source showed significant associations in case-control studies, in studies conducted in Ethiopia, and in health center–based settings, whereas no statistically significant associations were observed in cross-sectional studies, studies conducted in Indonesia, or across urban and rural populations. Similarly, water treatment was significantly associated with lower odds of stunting in rural and community-based settings, while no significant associations were identified in case-control and cross-sectional studies or in urban settings. For sanitation-related outcomes, sanitation facility type was significantly associated with lower odds of stunting across several subgroup categories, including case-control studies; studies conducted in Bangladesh, Ethiopia, Indonesia, and Mozambique; as well as in mixed (urban and rural) settings and health center–based studies. In contrast, no statistically significant associations were observed in cross-sectional studies, urban and rural populations, or community-based settings.

**Fig. 5:**
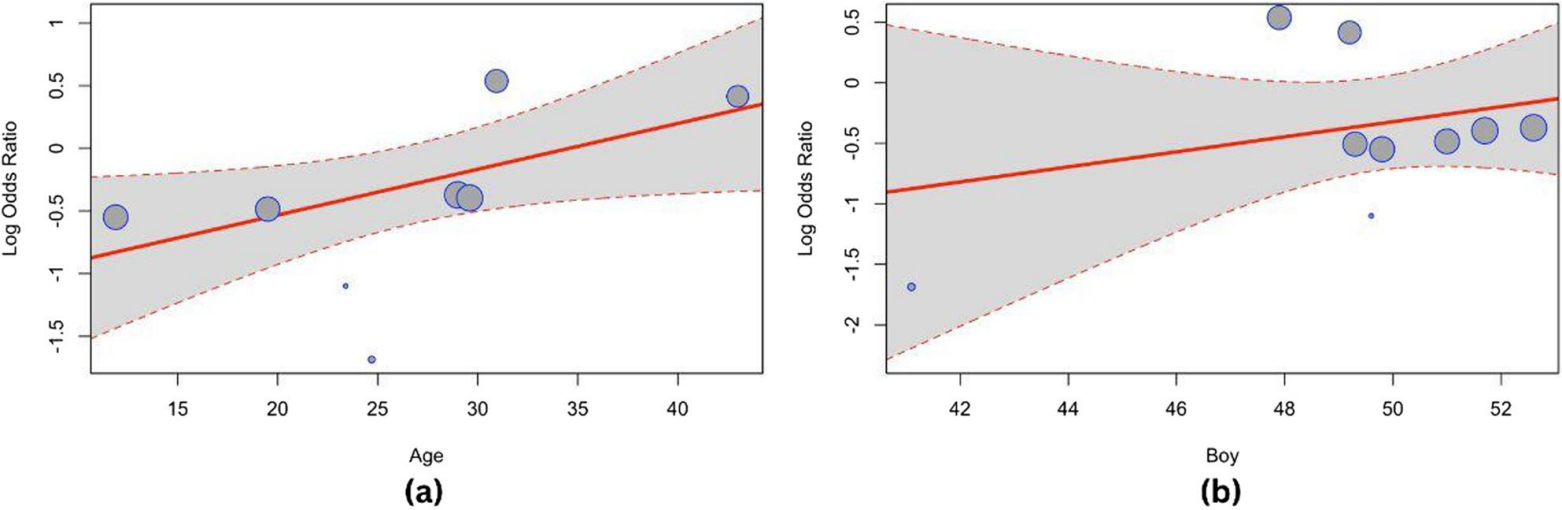
Meta-regression analysis of age (**a**) and proportion of boys (**b**) on the effect size of sanitation facility type

Meta-regression analysis further indicated that age was significantly associated with the effect size for sanitation facility type (*p* = 0.0407), whereas the proportion of boys was not (*p* = 0.4071), suggesting that age may partially explain the observed heterogeneity. These findings are illustrated in Fig. 5. Environmental sanitation demonstrated consistent significant associations with lower odds of stunting across all subgroup categories, including study design (case-control and cross-sectional), residence (urban and rural), study setting (community and health center), and study quality (good and fair). In contrast, safe disposal of feces showed significant associations only in studies conducted in Ethiopia, whereas no statistically significant associations were observed in studies conducted in Indonesia or across urban and rural populations, as well as community- and health center-based settings. For hygiene-related outcomes, hand hygiene was significantly associated with lower odds of stunting in studies conducted in Bangladesh and Ethiopia, as well as in mixed (urban and rural) settings, community-based studies, and studies of both good and fair quality. In contrast, no statistically significant associations were observed in exclusively urban or rural populations or in health center-based studies. Family hygiene did not demonstrate statistically significant associations across subgroup analyses, including urban, rural, community-based, and health center-based settings. General hygiene was significantly associated with lower odds of stunting in studies conducted in Ethiopia and Indonesia, whereas no statistically significant associations were observed in other subgroup categories, including those conducted in Mozambique.

### Publication bias

#### Publication bias of water outcome

**Fig. 6:**
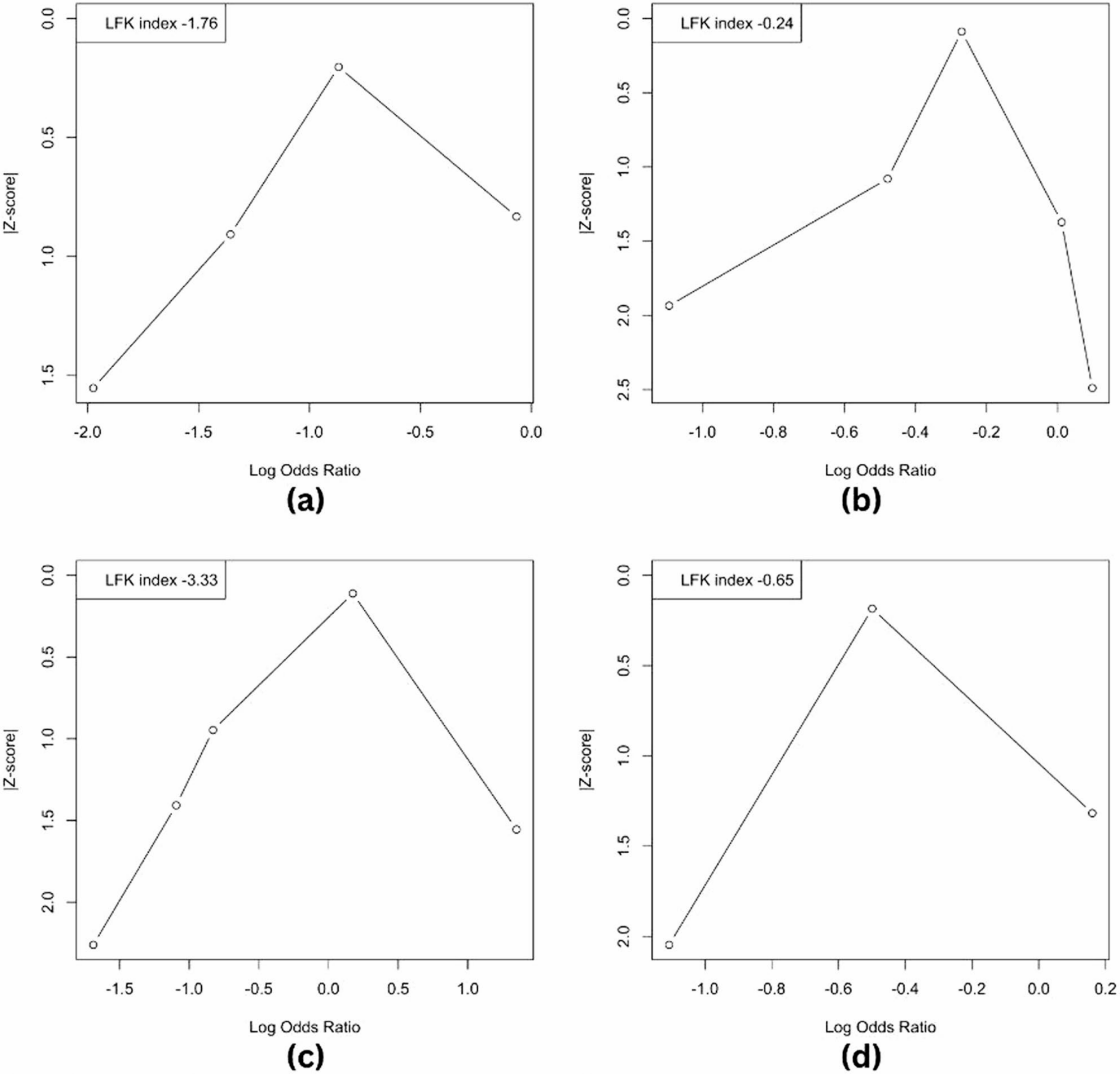
Publication bias assessed using DOI plots for drinking water quality (**a**), water accessibility (**b**), drinking water source (**c**), and water treatment (**d**)

The publication bias assessed using DOI plot for water outcome were presented in Fig. 6. The DOI plot for drinking water quality showed minor asymmetry, with an LFK index of -1.76, indicating a low risk of publication bias. Water accessibility and water treatment demonstrated no asymmetry, with LFK indices of -0.24 and − 0.65, respectively, also suggesting a low risk of publication bias. In contrast, water accessibility revealed major asymmetry, with an LFK index of -3.33, indicating a high risk of publication bias.

#### Publication bias of sanitation outcome

**Fig. 7:**
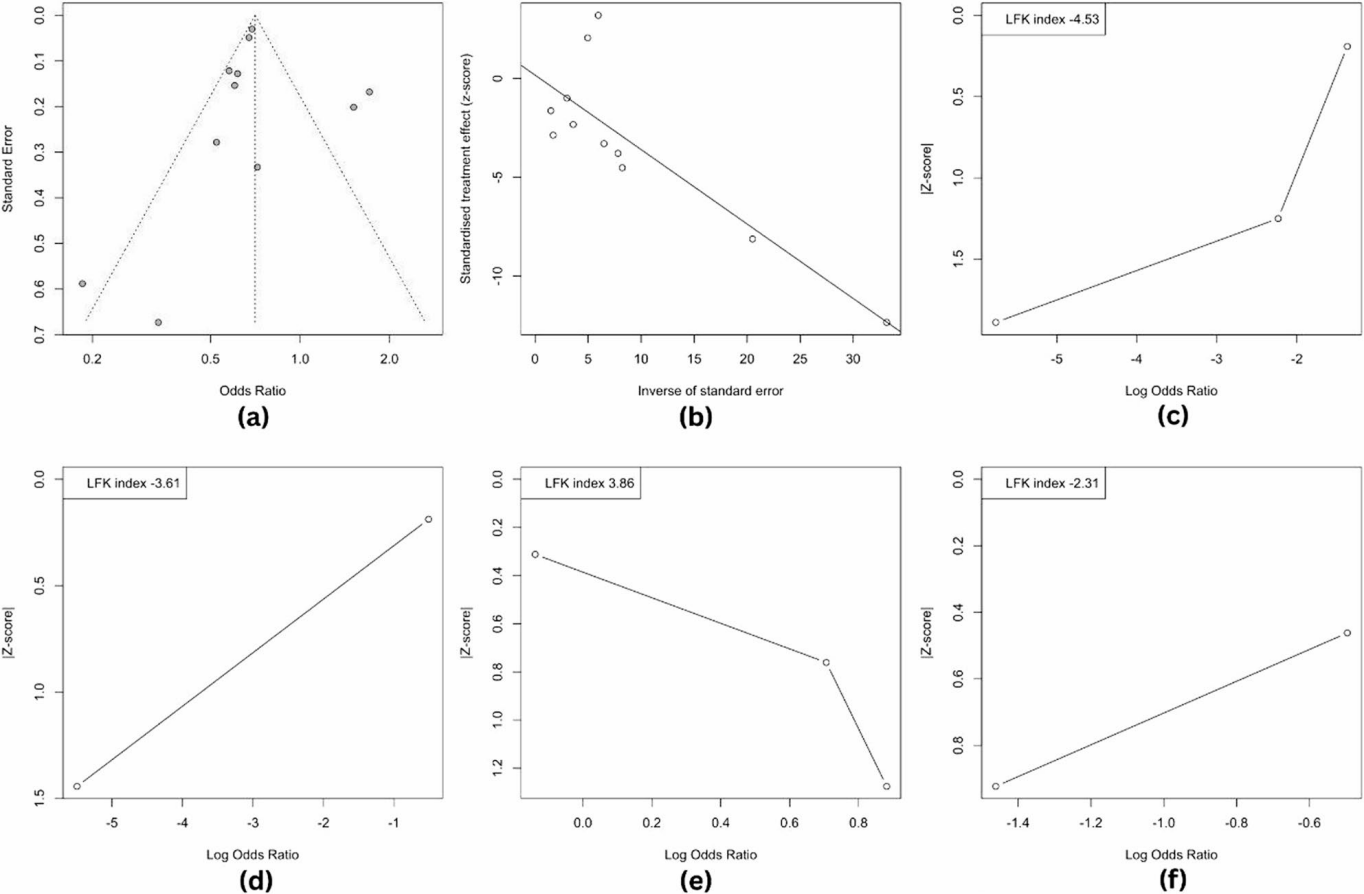
Publication bias assessed using funnel plot and Egger’s plot for sanitation facility type (**a**, **b**) and DOI plots for environmental sanitation (**c**), toilet access (**d**), safe disposal of feces (**e**), and garbage disposal (**f**) on childhood stunting

The publication bias was assessed using funnel plot and egger’s plot for drinking water quality and DOI plot for water accessibility, drinking water source, and water treatment were presented in Fig. 7. A funnel plot for drinking water quality showed that no visual asymmetry and Egger’s regression test indicated no evidence of small-study effects (*p* = 0.8724). DOI plots for environmental sanitation, toilet access, safe disposal of feces, and garbage disposal revealed major asymmetry with an LFK indices − 4.53, -3.61, 3.86, and − 2.31, respectively, indicating a high risk of publication bias.

#### Publication bias of hygiene outcome

**Fig. 8:**
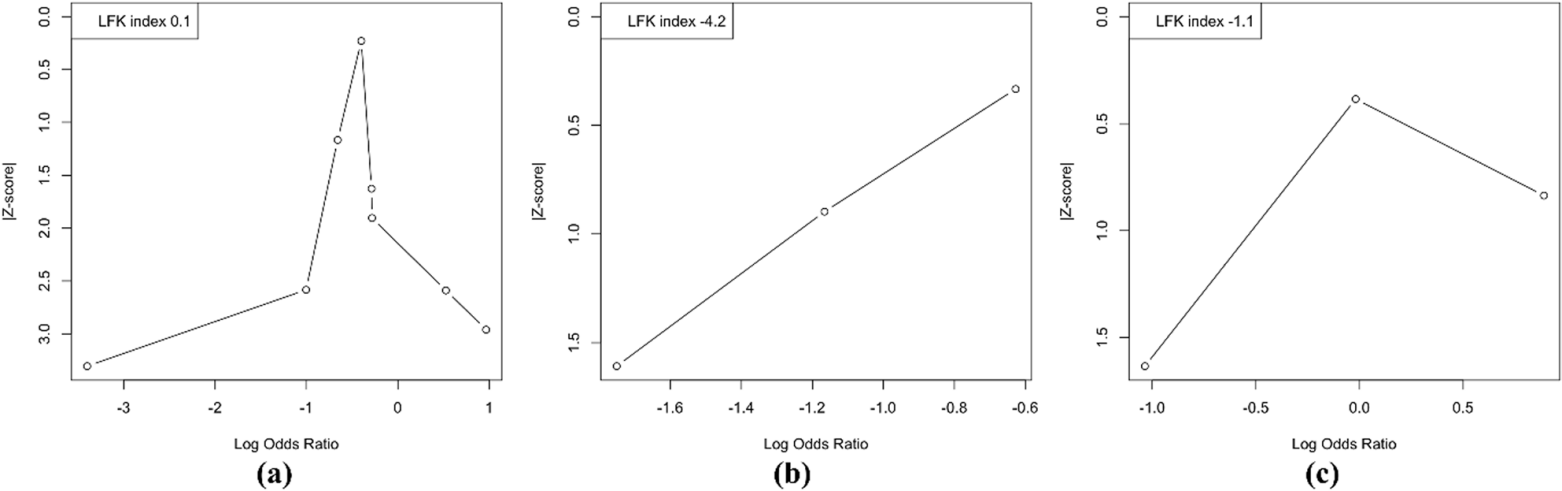
Publication bias assessed using DOI plots for hand hygiene (**a**), family hygiene (**b**), and general hygiene (**c**)

The publication bias assessed using DOI plot for hygiene outcome were presented in Fig. 8. The DOI plots for hand hygiene and general hygiene showed no asymmetry and minor asymmetry, with LFK indices of 0.1 and − 1.1, respectively, indicating a low risk of publication bias. In contrast, the DOI plot for family hygiene revealed major asymmetry, with an LFK index of -4.2, suggesting a high risk of publication bias.

### Sensitivity analysis

#### Sensitivity analysis of water outcome

Forest plots following sensitivity analyses for water outcomes are presented in Supplementary Fig. 1. For drinking water quality, omitting *Sapriyansyah et al.*,* 2024* substantially reduced heterogeneity from 61.6% to 6.0%, with the pooled estimate indicating significantly lower odds of stunting (OR = 0.29; 95% CI: 0.15–0.56; *p* = 0.0003). Similarly, for water accessibility, exclusion of *Hasan et al.*,* 2023 (MICS)* reduced heterogeneity from 62.5% to 24.3%, with the pooled estimate remaining significantly associated with lower odds of stunting (OR = 0.78; 95% CI: 0.64–0.95; *p* = 0.0142). In contrast, for water source, omitting *Ademas et al.*,* 2021* reduced heterogeneity from 91.0% to 87.4%; however, substantial heterogeneity persisted, and the pooled estimate was not statistically significant (OR = 0.48; 95% CI: 0.22–1.05; *p* = 0.0663).

#### Sensitivity analysis of sanitation outcome

Forest plots following sensitivity analyses for sanitation outcomes are presented in Supplementary Fig. 2. For sanitation facility type, omitting *Ademas et al.*,* 2021* reduced heterogeneity from 82.0% to 65.4%, while the pooled estimate remained significantly associated with lower odds of stunting (OR = 0.66; 95% CI: 0.54–0.81; *p* < 0.0001). For environmental sanitation, exclusion of *Sapriyansyah et al.*,* 2024* reduced heterogeneity from 92.1% to 30.1%, with the pooled estimate indicating significantly lower odds of stunting (OR = 0.21; 95% CI: 0.10–0.42; *p* < 0.0001). For safe disposal of feces, exclusion of *Torlesse et al.*,* 2016* substantially reduced heterogeneity from 87.7% to 0%, with the pooled estimate indicating significantly higher odds of stunting (OR = 2.34; 95% CI: 1.54–3.56; *p* < 0.0001), suggesting that the result is sensitive to the inclusion of this study.

#### Sensitivity analysis of hygiene outcome

Forest plots following sensitivity analyses for hygiene outcomes are presented in Supplementary Fig. 3. For hand hygiene, omitting *Hasan et al.*,* 2023 (BDHS)* reduced heterogeneity from 85.9% to 79.1%; however, considerable heterogeneity persisted, and the pooled estimate was not statistically significant (OR = 0.52; 95% CI: 0.23–1.19; *p* = 0.1205). For general hygiene, exclusion of *Ademas et al.*,* 2021* reduced heterogeneity from 93.7% to 86.7%. Despite this reduction, substantial heterogeneity remained, and the pooled estimate was not statistically significant (OR = 0.62; 95% CI: 0.23–1.66; *p* = 0.3400).

## Discussion

This systematic review and meta-analysis demonstrated that several dimensions of WASH practices are associated with reduced odds of childhood stunting in LMICs. Our results suggested that improvements in several WASH components, particularly water quality, water accessibility, sanitation facility type, environmental sanitation, hand hygiene, and family hygiene, are significantly associated with reduced odds of childhood stunting. However, not all WASH components showed significant effects, particularly drinking water source, water treatment, toilet access, safe disposal of child feces, garbage disposal, and general hygiene practices.

Subgroup analyses indicated that associations were context-dependent. For water-related outcomes, drinking water quality was more strongly associated with lower odds of stunting in urban, community-based, and higher-quality studies, but not in rural or health center-based settings. Water accessibility showed similar patterns across several study designs and rural or mixed settings, although findings were inconsistent across countries and urban populations. Drinking water source and water treatment showed inconsistent associations across subgroups. For sanitation-related outcomes, sanitation facility type was associated with lower odds of stunting in several subgroups, including case-control studies and multiple country settings, but these findings were not consistent across all study designs and populations, reflecting substantial heterogeneity.

Sensitivity analyses revealed that several findings were influenced by individual studies. While some associations, such as water quality and sanitation facility type, remained directionally consistent after study exclusion, others were less stable. In particular, the association for hand hygiene lost statistical significance after removal of a single large study [29], and the association for safe disposal of feces changed in magnitude and direction, indicating substantial instability [37]. For sanitation facility type, the association remained statistically significant after exclusion of one study, although substantial heterogeneity persisted [25] indicating limited robustness of the pooled estimate. Publication bias assessments further indicated potential small-study effects in several outcomes, particularly for water accessibility, environmental sanitation, toilet access, safe disposal of feces, garbage disposal, and family hygiene, where substantial asymmetry was observed, while other outcomes showed low or minimal evidence of bias. Moreover, several pooled estimates were accompanied by substantial or very high heterogeneity. Accordingly, these findings should be interpreted as observational associations rather than definitive causal targets for intervention.

Several biologically plausible mechanisms may explain the observed associations between poor WASH conditions and childhood stunting, particularly through pathways involving enteric infection, gastrointestinal dysfunction, and impaired nutrient utilization. Exposure to fecally contaminated water, such as *Escherichia coli* contamination, has been shown to increase the probability of stunting, with diarrhoeal illness identified as a key mediating mechanism linking pathogen exposure to impaired growth [39]. Beyond acute diarrhoea, chronic and repeated exposure to enteric pathogens may contribute to environmental enteric dysfunction, characterized by intestinal inflammation, increased permeability, and reduced absorptive capacity. This condition disrupts nutrient uptake and has been consistently associated with impaired linear growth and undernutrition in young children [40]. In addition, parasitic infections, particularly soil-transmitted helminths, may further exacerbate nutritional deficits by altering gut microbiota composition, promoting inflammation, and interfering with nutrient metabolism, thereby compounding growth impairment [41]. These interrelated processes highlight a complex infection–malnutrition cycle in which poor WASH conditions facilitate repeated pathogen exposure, leading to both clinical and subclinical gastrointestinal disturbances that ultimately compromise child growth.

Children in households with higher quality drinking water had significantly lower odds of stunting, suggesting that reducing exposure to contaminated water may be associated with a lower likelihood of chronic undernutrition. Moreover, adequate access to water may facilitate improved hygiene practices, which in turn may reduce infection burden by enabling its use for handwashing, food preparation, and regular child hygiene. These findings are consistent with a recent review reporting 27% lower odds of stunting due to access to improved water [42]. However, the type of water source was not significantly associated with stunting. Previous analysis in Indonesia reported no significant difference in stunting between households with improved and unimproved water sources [34]. Similarly, a multi-survey analysis in Ethiopia found that the source of drinking water did not predict stunting prevalence [43]. These findings imply that the quality and reliability of water may matter more than the source’s basic classification. Meanwhile, household water treatment was not significantly associated with stunting. This lack of effect could be due to inconsistent adoption of water treatment or insufficient effectiveness of treatment or recontamination during storage.

Children living in households with improved sanitation facilities had significantly lower odds of stunting. A previous meta-analysis reported that sanitation interventions reduced stunting risk by 15–23% [44]. However, prior analyses primarily focused on sanitation components alone, whereas the present study provides a more comprehensive evaluation across multiple WASH domains. A previous ecological study showed that an increase in the population practicing open defecation caused a rise in stunting rates [45]. On the other hand, improvement in environmental sanitation was associated with lower odds of stunting. Environmental sanitation reflects community-level sanitation conditions or open defecation rates in the area. Living in a community with widespread sanitation coverage, particularly minimal open defecation and proper waste management could reduce the stunting risk attributed to poor sanitation. Interestingly, some related sanitation practices did not show statistically significant effects on stunting in our analysis. Household toilet access was not associated with stunting reduction. In some contexts, having any toilet is somewhat better than none, but in other cases unimproved pit toilets may not confer much benefit over open defecation, especially if they are poorly maintained [44]. Garbage disposal practices were not significantly associated with stunting. Although poor solid waste management may increase environmental contamination through vector proliferation (e.g., flies and rodents), its contribution to chronic undernutrition appears less direct compared with pathways involving fecal contamination [46]. Flies, for instance, can mechanically carry enteric pathogens from waste to food preparation areas, while rodents contribute to food contamination and can harbor pathogens such as *Salmonella enterica*, *Escherichia coli*, and *Campylobacter* spp [47].

Safe disposal of children’s feces was not associated with lower stunting in the main analysis. This is counterintuitive since young children’s feces are known to carry pathogens and unsafe disposal could contaminate the household environment. This could be due to high heterogeneity. Thereby, after performing sensitivity analysis, the heterogeneity dropped substantially with results indicating higher odds of stunting associated with unsafe feces disposal. This finding suggests that inappropriate disposal of children’s feces may meaningfully increase exposure to enteric pathogens when consistently measured. Young children’s feces often contain higher pathogen loads than adult feces and are more likely to contaminate household environments due to unsafe disposal practices such as open defecation or disposal in open garbage [48].

Hygiene practices were associated with reduced odds of stunting, particularly through improvements in personal and household hygiene. Hand hygiene was linked to substantially lower odds of stunting, suggesting that children in families practicing good hand hygiene had lower risk of stunting compared to those in households with poor hygiene. Handwashing with soap can prevent the spread of pathogens and environmental enteric bacteria within the household, thereby reducing the frequency of illnesses that impair growth [49]. On the other hand, family hygiene, reflecting overall personal cleanliness of household members and food hygiene, was also associated with lower stunting. Our meta-analysis found that general hygiene was not significantly associated with stunting. This was particularly due to a broad category of hygiene with no specific hygiene behaviors representing measurable health benefits.

While our findings further support the role of WASH in relation to childhood stunting, several important limitations must be acknowledged. All included studies were observational, many were cross-sectional, and the pooled analyses were based on crude event data rather than consistently adjusted effect estimates. Accordingly, causal inference cannot be established, and residual confounding is likely. In addition, the definitions of WASH exposures varied substantially across studies, which likely contributed to the observed heterogeneity and may have introduced misclassification. Some outcomes were also informed by a limited number of studies, reducing precision and making subgroup findings especially vulnerable to instability.

This meta-analysis reinforces that achieving SDG 2 (“Zero Hunger”) and SDG 6 (“Clean Water and Sanitation”) will require integrated, behavior-driven WASH strategies rather than infrastructure expansion alone. Sustainable reductions in childhood stunting may be difficult to achieve without consistent water quality assurance, safe excreta management, and sustained community-level hygiene promotion. These findings emphasize that quality, behavioral uptake, and environmental context are as critical as access itself. Future research should prioritize standardized WASH metrics, objective exposure assessment particularly for child feces management and longitudinal or intervention-based designs capable of clarifying causal pathways. Integrative approaches examining the interaction between WASH, nutrition, enteric infections, and environmental enteric dysfunction will be essential to inform multisectoral policies aimed at accelerating stunting reduction in LMICs.

## Conclusion

This meta-analysis indicates that several WASH indicators were associated with lower odds of childhood stunting in observational studies from LMICs. Drinking water quality, water accessibility, and sanitation facility type were more consistently associated with lower odds of stunting, whereas environmental sanitation, hand hygiene, and safe disposal of child feces showed inconsistent findings. Other WASH components did not demonstrate consistent associations. Overall, the certainty of evidence is limited by heterogeneity, observational study design, and instability in some analyses. These findings should therefore be interpreted as indicative rather than confirmatory, and further well-designed studies are needed to clarify causal relationships.

## Supplementary Information

Supplementary Material 1.

## Data Availability

The datasets used and/or analyzed during the current study are available from the corresponding author on reasonable request. All data were derived from previously published studies, which are cited in the reference list.
