# Supplementary material for: Association between water, sanitation, and hygiene (WASH) practices and childhood stunting in low- and middle-income countries: a systematic review and meta-analysis
Source: BMC Public Health. 2026 May 5;26:1960. doi: 10.1186/s12889-026-27639-z (PMC13312774; doi:10.1186/s12889-026-27639-z)
Supplement: Supplementary file 1 — Supplementary Material 1. [file 12889_2026_27639_MOESM1_ESM.docx]

**Supplementary Material**

**Supplementary Table 1.** Keywords combination

| Database | Keywords combination | Hits |
| --- | --- | --- |
| PubMed | ((((((((((((((((child*[Title/Abstract]) OR (baby[Title/Abstract])) OR (babies[Title/Abstract])) OR (infant*[Title/Abstract])) OR (toddler*[Title/Abstract])) OR (preschool[Title/Abstract])) OR (pediatric*[Title/Abstract])) OR (paediatric*[Title/Abstract])) OR ("under five"[Title/Abstract])) OR (under-five[Title/Abstract])) OR (under-5[Title/Abstract])) OR ("under 5"[Title/Abstract])) OR (Child[MeSH Terms])) OR (Infant[MeSH Terms])) OR (Pediatrics[MeSH Terms])) AND (((((stunting[Title/Abstract]) OR ("growth curve"[Title/Abstract])) OR ("Growth Disorder"[Title/Abstract])) OR ("Stunted Growth"[Title/Abstract])) OR (stunting[MeSH Terms]))) AND (((((((Water[Title/Abstract]) OR (Sanitation[Title/Abstract])) OR (Hygiene[Title/Abstract])) OR (WASH[Title/Abstract])) OR (Water[MeSH Terms])) OR (Sanitation[MeSH Terms])) OR (Hygiene[MeSH Terms])) | 1,123 |
| Scopus | ( TITLE-ABS-KEY ( child* OR baby OR babies OR infant* OR toddler* OR preschool OR pediatric* OR paediatric* OR "under five" OR under-five OR under-5 OR "under 5" ) ) AND ( TITLE-ABS-KEY ( stunting* OR "growth curve" OR "Growth Disorder" OR "Stunted Growth" ) ) AND ( TITLE-ABS-KEY ( Water OR Sanitation OR Hygiene OR WASH ) ) | 1,935 |
| Web of Science | ((TS=(child* OR baby OR babies OR infant* OR toddler* OR preschool OR pediatric* OR paediatric* OR "under five" OR under-five OR under-5 OR "under 5")) AND TS=(stunting* OR “growth curve” OR "Growth Disorder" OR "Stunted Growth")) AND TS=(Water OR Sanitation OR Hygiene OR WASH) | 1,156 |
| EBSCOhost | TI (child* OR baby OR babies OR infant* OR toddler* OR preschool OR pediatric* OR paediatric* OR "under five" OR under-five OR under-5 OR "under 5") AND TI (stunting* OR “growth curve” OR "Growth Disorder" OR "Stunted Growth") AND TI (Water OR Sanitation OR Hygiene OR WASH) | 18 |
| Cochrane Library | (child* OR baby OR babies OR infant* OR toddler* OR preschool OR pediatric* OR paediatric* OR "under five" OR under-five OR under-5 OR "under 5"):ti,ab,kw AND (stunting* OR “growth curve” OR "Growth Disorder" OR "Stunted Growth"):ti,ab,kw AND (Water OR Sanitation OR Hygiene OR WASH):ti,ab,kw | 176 |
| Epistemonikos | (title:(child* OR baby OR babies OR infant* OR toddler* OR preschool OR pediatric* OR paediatric* OR "under five" OR under-five OR under-5 OR "under 5") OR abstract:(child* OR baby OR babies OR infant* OR toddler* OR preschool OR pediatric* OR paediatric* OR "under five" OR under-five OR under-5 OR "under 5")) AND (title:(stunting* OR "growth curve" OR "Growth Disorder" OR "Stunted Growth") OR abstract:(stunting* OR "growth curve" OR "Growth Disorder" OR "Stunted Growth")) AND (title:(Water OR Sanitation OR Hygiene OR WASH) OR abstract:(Water OR Sanitation OR Hygiene OR WASH)) | 420 |
| Google Scholar | (child* OR baby OR babies OR infant* OR toddler* OR preschool OR pediatric* OR paediatric* OR "under five" OR under-five OR under-5 OR "under 5") AND (stunting* OR “growth curve” OR "Growth Disorder" OR "Stunted Growth") AND (Water OR Sanitation OR Hygiene OR WASH) | 8701 |

**Supplementary Figure 1.** Forest plots after sensitivity analysis for water outcomes, including drinking water quality (a), water accessibility (b), and water source (c)


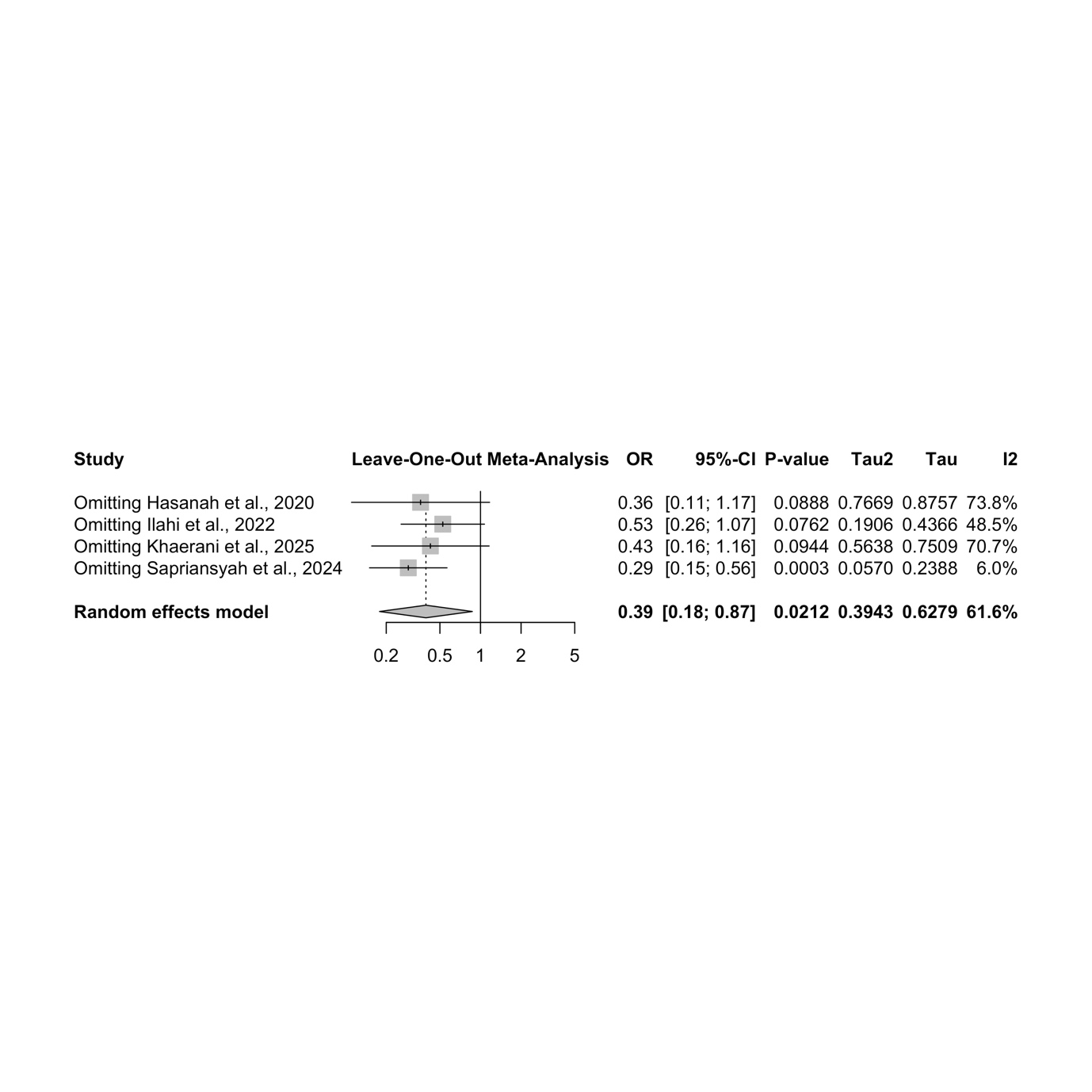


**(a)**

**
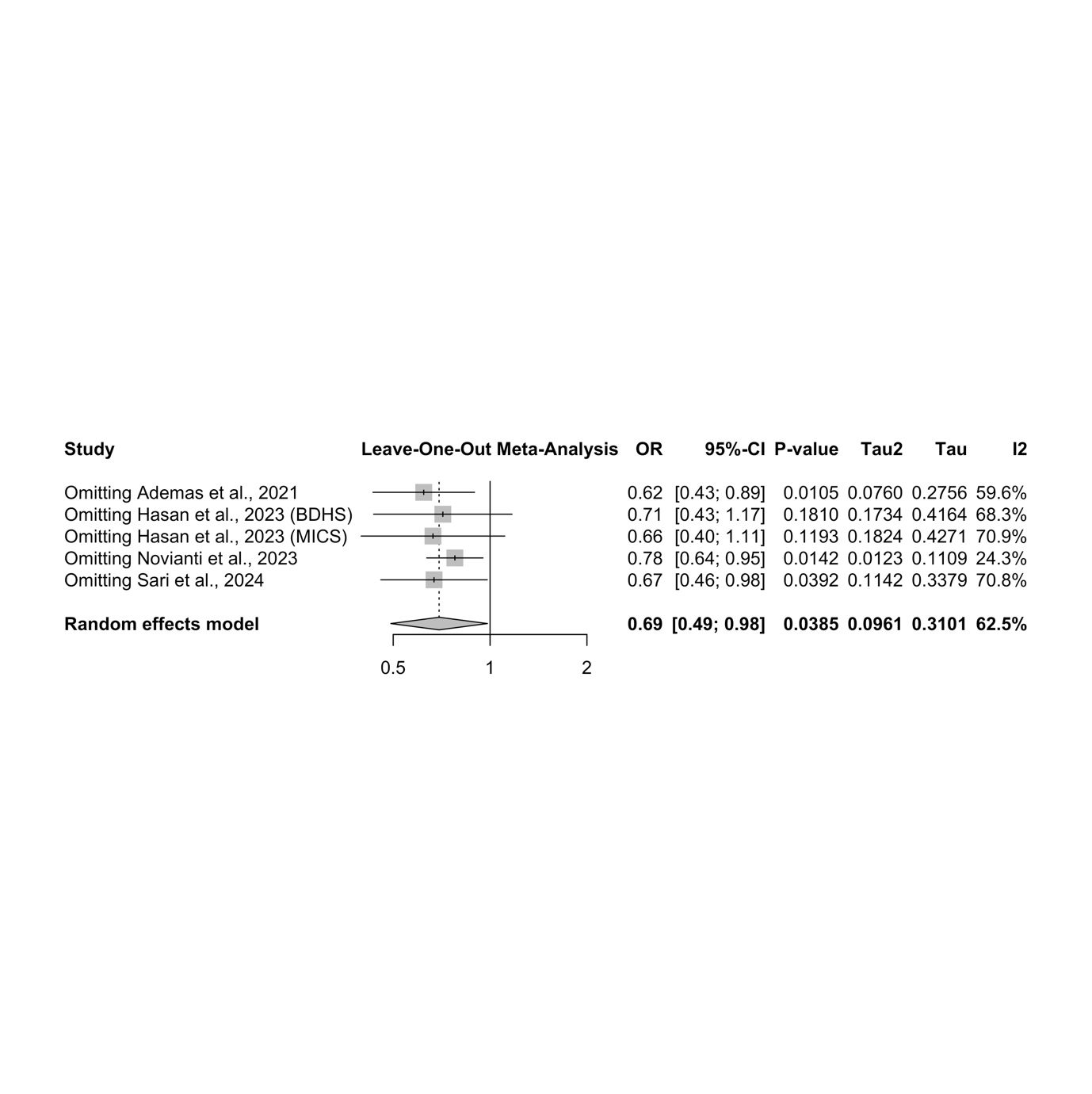
**

**(b)**

**
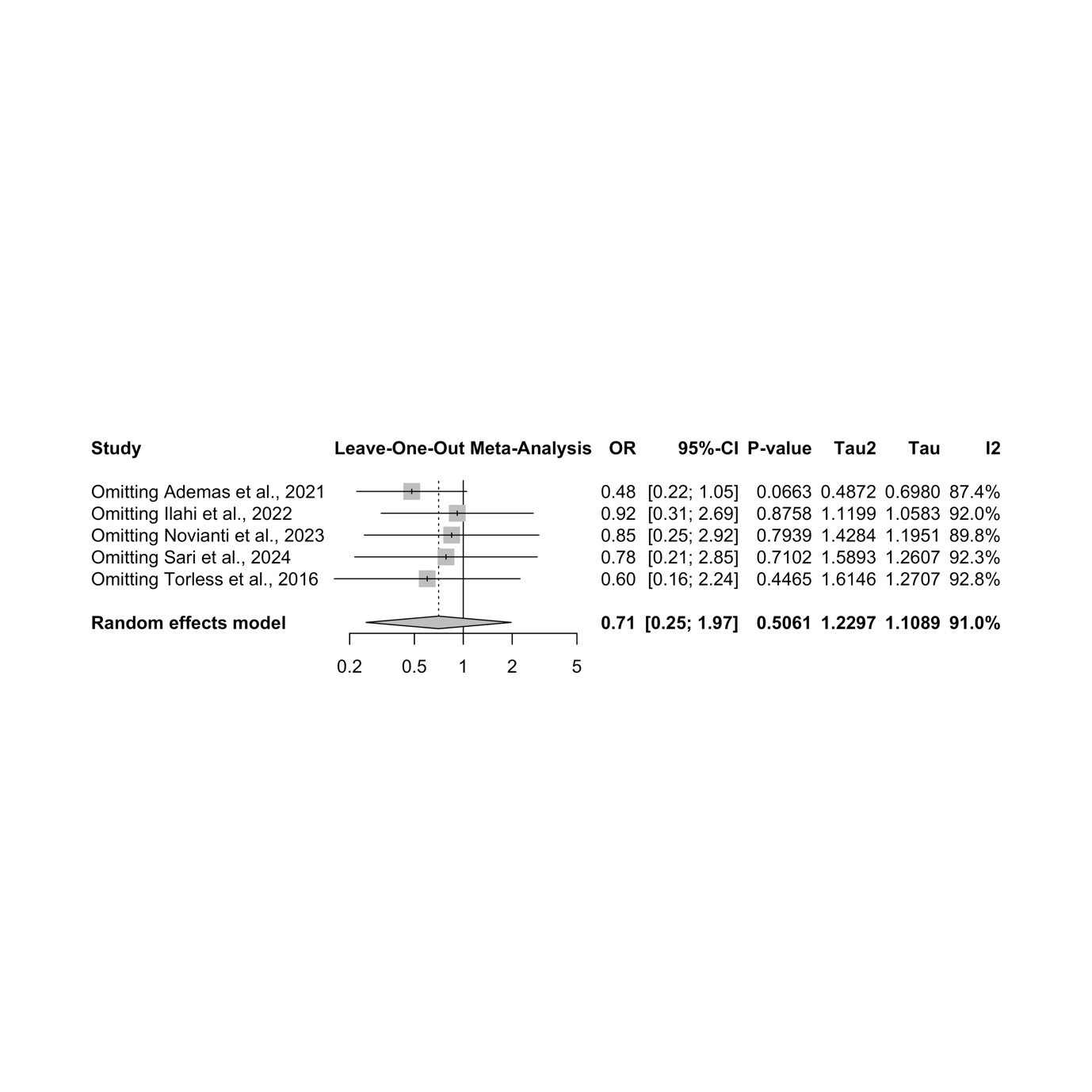
**

**(c)**

**Supplementary Figure 2.** Forest plot after sensitivity analysis for sanitation outcome, including sanitation facility type (a), environmental sanitation (b), and safe disposal feces (c)


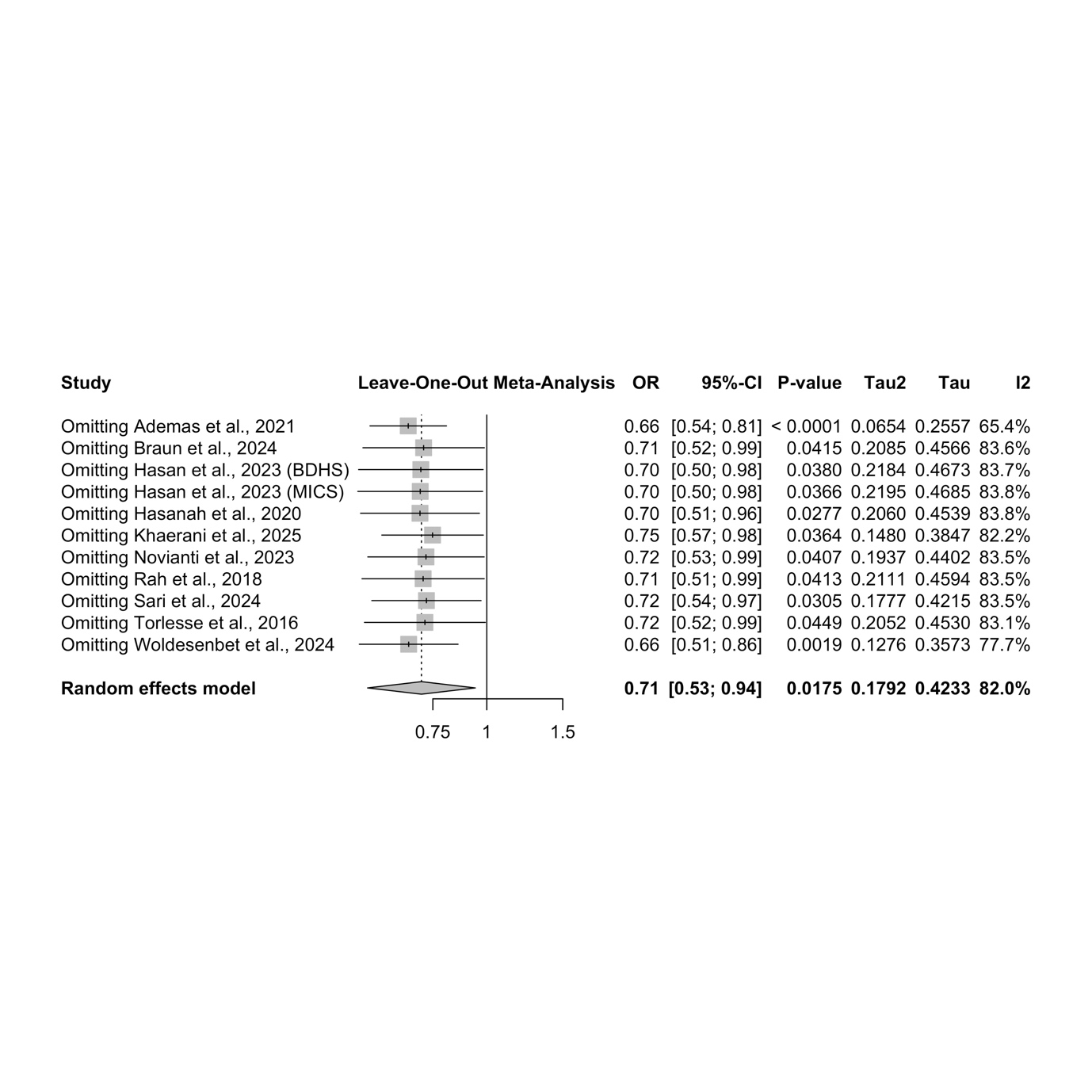


**(a)**

**
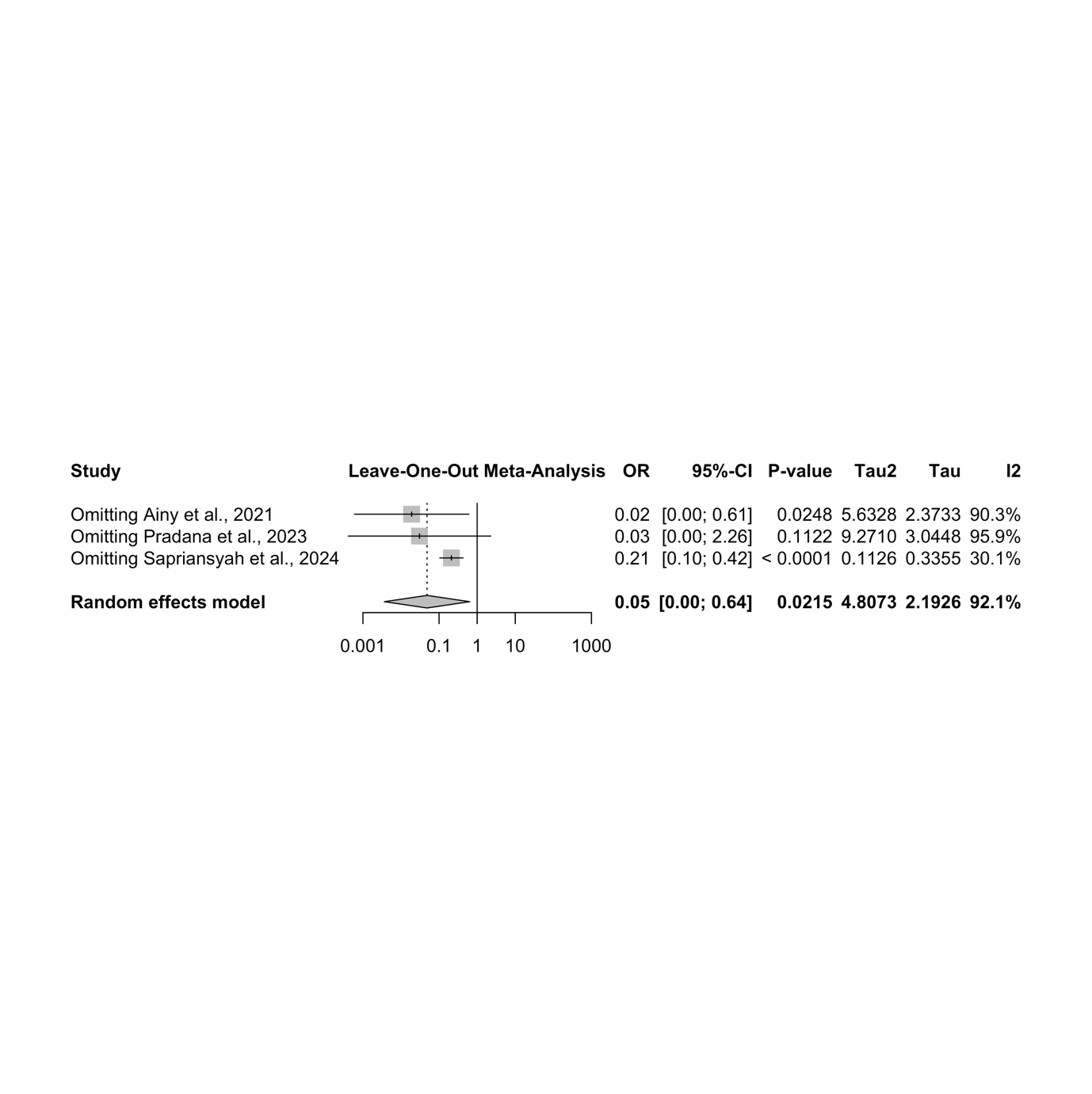
**

**(b)**

**
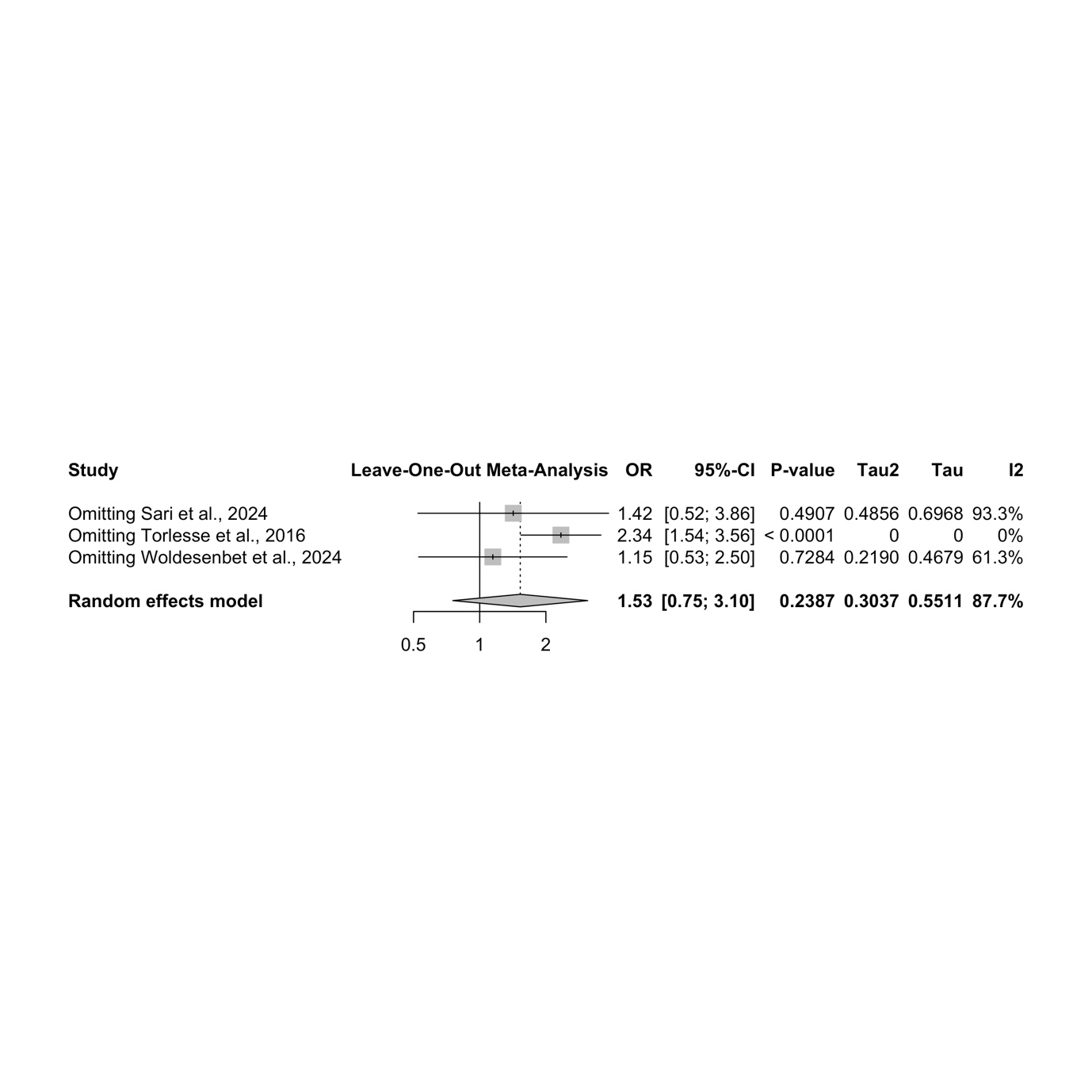
**

**(c)**

**Supplementary Figure 3.** Forest plot after sensitivity analysis for hygiene outcome, including hand hygiene (a) and general hygiene (b)


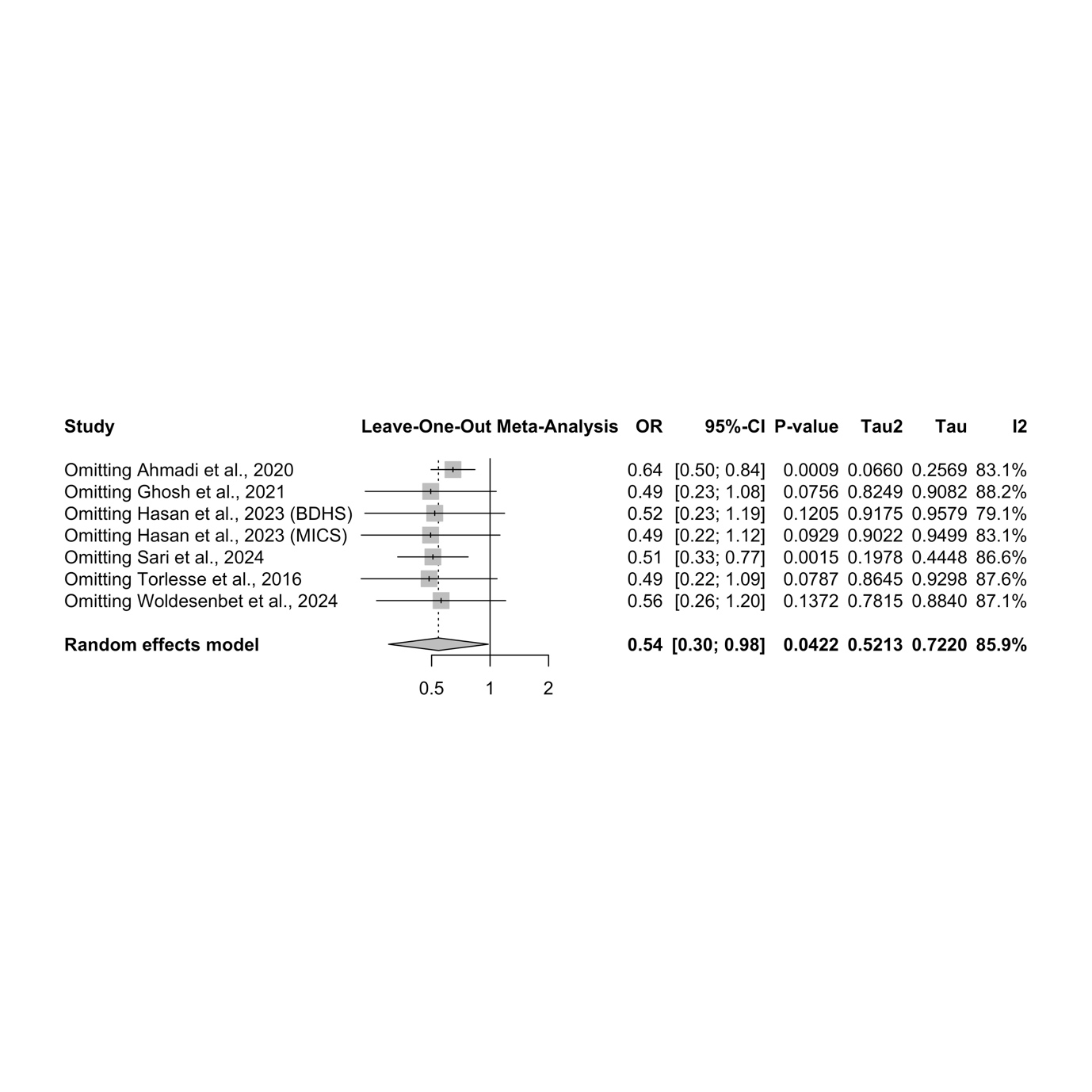


**(a)**

**
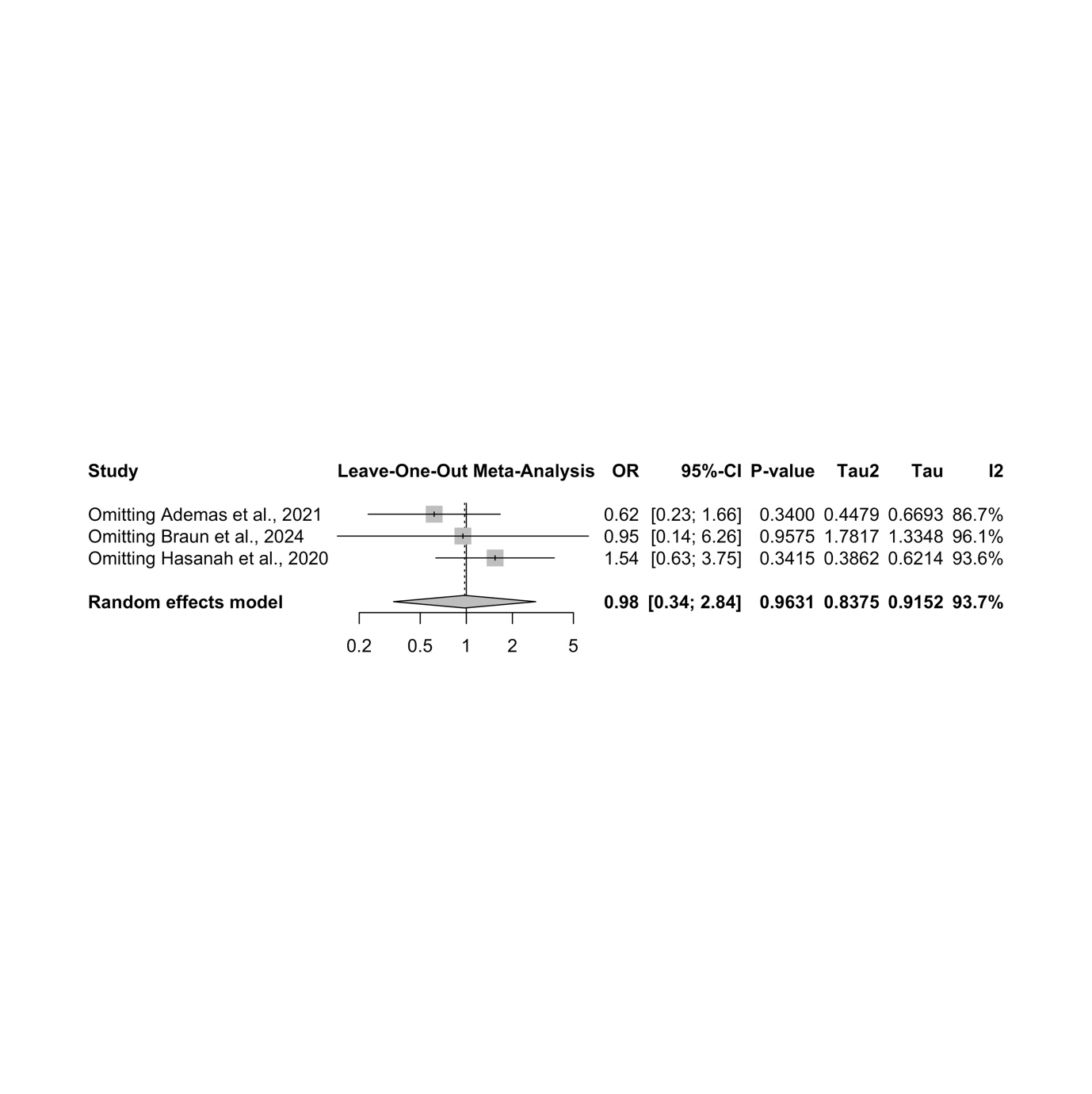
**

**(b)**

**Supplementary Table 2.** Subgroup analysis

| **Variable** | **k** | **OR (95%CI)** | ***p-value*** | **I^2^** |
| --- | --- | --- | --- | --- |
| **Drinking water quality** | | | | |
| Urban  Rural | 2  2 | 0.27 (0.10; 0.79)  0.55 (0.16; 1.92) | 0.0163*  0.3501 | 51.3%  65.4% |
| Community  Health center | 2  2 | 0.27 (0.10; 0.79)  0.55 (0.16; 1.92) | 0.0163*  0.3501 | 51.3%  65.4% |
| Quality good  Quality fair | 3  1 | 0.29 (0.15; 0.56)  0.39 (0.18; 0.87) | 0.0003*  0.8551 | 6%  - |
| **Water accessibility** | | | | |
| Case-control  Cross-sectional | 1  4 | 0.33 (0.18; 0.62)  0.78 (0.64; 0.95) | 0.0005*  0.0142* | -  24.3% |
| Bangladesh  Ethiopia  Indonesia | 2  1  2 | 0.72 (0.61; 0.86)  1.01 (0.70; 1.47)  0.53 (0.17; 1.66) | 0.0004*  0.9529  0.2787 | 11.8%  -  - |
| Urban  Rural  Mix (urban + rural) | 2  1  2 | 1.02 (0.71; 1.45)  0.33 (0.18; 0.62)  0.72 (0.61; 0.86) | 0.9205  0.0005*  0.0004* | 0%  -  11.8% |
| Community  Health center | 3  2 | 0.77 (0.62; 0.95)  0.53 (0.17; 1.66) | 0.0159*  0.2787 | 45.2%  64.1% |
| **Drinking water source** | | | | |
| Case-control  Cross-sectional | 2  3 | 0.30 (0.17; 0.53)  1.28 (0.39; 4.24) | <0.0001*  0.6857 | 0%  90.6% |
| Ethiopia  Indonesia | 1  4 | 3.85 (2.13; 6.97)  0.48 (0.22; 1.05) | <0.0001*  0.0663 | -  87.4% |
| Urban  Rural | 3  2 | 0.72 (0.12; 2.49)  0.65 (0.19; 2.26) | 0.7209  0.4994 | 93.2%  92.9% |
| Community  Health center | 3  2 | 1.03 (0.20; 5.35)  0.37 (0.23; 0.60) | 0.9678        <0.0001* | 90.8%  0% |
| **Water treatment** | | | | |
| Case-control  Cross-sectional | 1  2 | 0.33 (0.07; 1.63)  0.78 (0.42; 1.45) | 0.1731  0.4281 | -  58.5% |
| Urban  Rural | 2  1 | 0.76 (0.24; 2.48)  0.61 (0.42; 0.88) | 0.6526  0.0078* | 49.9%  - |
| Community  Health center | 2  1 | 0.59 (0.41; 0.84)  1.17 (0.56; 2.48) | 0.0038*  0.6740 | 0%  - |
| **Sanitation facility type** | | | | |
| Case-control  Cross-sectional | 3  8 | 0.49 (0.27; 0.87)  0.78 (0.57; 1.07) | 0.0142*  0.1245 | 50.3%  85.9% |
| Bangladesh  Ethiopia  Indonesia  Mozambique | 2  2  6  1 | 0.68 (0.65; 0.72)  1.63 (1.26; 2.10)  0.58 (0.49; 0.68)  0.60 (0.45; 0.81) | <0.0001*  0.0002*  <0.0001*  0.0010* | 0%  0%  4.1%  - |
| Urban  Rural  Mix (urban + rural) | 4  5  2 | 0.80 (0.42; 1.51)  0.62 (0.37; 1.06)  0.68 (0.65; 0.72) | 0.4885  0.0814  <0.0001* | 87.5%  83.2%  0% |
| Community  Health center | 8  3 | 0.80 (0.60; 1.07)  0.37 (0.19; 0.72) | 0.1333  0.0032* | 85.5%  25.3% |
| **Environmental sanitation** | | | | |
| Case-control  Cross-sectional | 2  1 | 0.02 (0.00; 0.61)  0.25 (0.16; 0.40) | 0.0248*  <0.0001* | 90.3%  - |
| Urban  Rural | 1  2 | 0.25 (0.16; 0.40)  0.02 (0.00; 0.61) | <0.0001*  0.0248* | -  90.3% |
| Community  Health center | 1  2 | 0.25 (0.16; 0.40)  0.02 (0.00; 0.61) | <0.0001*  0.0248* | -  90.3% |
| Quality good  Quality fair | 2  1 | 0.21 (0.10; 0.42)  0.00 (0.00; 0.02) | <0.0001*  <0.0001* | 30.1%  - |
| **Safe disposal feces** | | | | |
| Ethiopia  Indonesia | 1  2 | 2.41 (1.52; 3.83)  1.15 (0.53; 2.50) | 0.0002*  0.7284 | -  61.3% |
| Urban  Rural | 1  2 | 2.03 (0.74; 5.52)  1.42 (0.52; 3.36) | 0.1673  0.4907 | -  93.3% |
| Community  Health center | 1  2 | 2.03 (0.74; 5.52)  1.42 (0.52; 3.36) | 0.1673  0.4907 | -  93.3% |
| **Hand hygiene** | | | | |
| Bangladesh  Ethiopia  Indonesia | 3  1  3 | 0.60 (0.48; 0.75)  0.37 (0.21; 0.64)  0.39 (0.04; 3.59) | <0.0001*  0.0004*  0.4035 | 89.1%  -  89.1% |
| Urban  Rural  Mix (urban + rural) | 1  4  2 | 1.69 (0.74; 3.90)  0.34 (0.10; 1.16)  0.59 (0.46; 0.76) | 0.2158  0.0844  <0.0001* | -  84.2%  94.5% |
| Community  Health center | 6  1 | 0.51 (0.33; 0.77)  1.69 (0.74; 3.90) | 0.0015*  0.2158 | 86.6%  - |
| Quality good  Quality fair | 6  1 | 0.64 (0.50; 0.84)  0.03 (0.01; 0.16) | 0.0009*  <0.0001* | 83.1%  - |
| **Family hygiene** | | | | |
| Urban  Rural | 1  2 | 0.31 (0.09; 1.05)  0.37 (0.13; 1.04) | 0.0603  0.0600 | -  50.9% |
| Community  Health center | 1  2 | 0.31 (0.09; 1.05)  0.37 (0.13; 1.04) | 0.0603  0.0600 | -  50.9% |
| **General hygiene** | | | | |
| Ethiopia  Indonesia  Mozambique | 1  1  1 | 2.43 (1.74; 3.40)  0.35 (0.18; 0.69)  0.98 (0.72; 1.33) | <0.0001*  0.0022*  0.9010 | -  -  - |

*p<0.05 statistically significant. k=number of study.
